# Non-alcoholic fatty liver disease in hemochromatosis probands with iron overload and *HFE* p.C282Y/p.C282Y

**DOI:** 10.1186/s12876-023-02763-x

**Published:** 2023-04-28

**Authors:** James C. Barton, J. Clayborn Barton, Ronald T. Acton

**Affiliations:** 1 Department of Medicine, University of Alabama at Birmingham, Birmingham, AL USA; 2 Southern Iron Disorders Center, Birmingham, AL USA; 3 Department of Microbiology, University of Alabama at Birmingham, Birmingham, AL USA

**Keywords:** Alanine aminotransferase, Aspartate aminotransferase, Metabolic syndrome, Obesity, Serum ferritin, Type 2 diabetes

## Abstract

**Background:**

The aim of this study was to identify characteristics of non-alcoholic fatty liver disease (NAFLD) in adults with *HFE* p.C282Y/p.C282Y.

**Methods:**

We retrospectively studied non-Hispanic white hemochromatosis probands with iron overload (serum ferritin (SF) > 300 µg/L (M), > 200 µg/L (F)) and p.C282Y/p.C282Y at non-screening diagnosis who did not report alcohol consumption > 14 g/d, have cirrhosis or other non-NAFLD liver disorders, use steatogenic medication, or have diagnoses of heritable disorders that increase NAFLD risk. We identified NAFLD-associated characteristics using univariate and multivariable analyses.

**Results:**

There were 66 probands (31 men, 35 women), mean age 49 ± 14 (SD) y, of whom 16 (24.2%) had NAFLD. The following characteristics were higher in probands with NAFLD: median SF (1118 µg/L (range 259, 2663) vs. 567 µg/L (247, 2385); p = 0.0192); prevalence of elevated ALT/AST (alanine/aspartate aminotransferase) (43.8% vs. 10.0%; p = 0.0056); and prevalence of type 2 diabetes (T2DM) (31.3% vs. 10.0%; p = 0.0427). Mean age, sex, and prevalences of human leukocyte antigen-A*03 positivity, body mass index ≥ 30.0 kg/m^2^, hyperlipidemia, hypertension, and metabolic syndrome in probands with/without NAFLD did not differ significantly. Logistic regression on NAFLD using variables SF, elevated ALT/AST, and T2DM revealed: SF (p = 0.0318; odds ratio 1.0–1.0) and T2DM (p = 0.0342; 1.1–22.3). Median iron removed to achieve iron depletion (QFe) in probands with/without NAFLD did not differ significantly (3.6 g (1.4–7.2 g) vs. 2.8 g (0.7–11.0 g), respectively; p = 0.6862).

**Conclusions:**

NAFLD in hemochromatosis probands with p.C282Y/p.C282Y is associated with higher median SF and greater T2DM prevalence, after adjustment for other factors. NAFLD does not influence QFe significantly.

**Supplementary Information:**

The online version contains supplementary material available at 10.1186/s12876-023-02763-x.

## Background

Non-alcoholic fatty liver disease (NAFLD) is a spectrum of liver abnormalities characterized by steatosis (lipid-filled vacuoles in hepatocytes), steatohepatitis (steatosis and hepatocyte “ballooning” with inflammation and necrosis), fibrosis, and cirrhosis [1, 2]. It has been estimated that 25.2% (95% confidence interval (CI) [22.1, 28.7]) of persons world-wide have NAFLD [2]. In a meta-analysis of 34 studies, the prevalence of NAFLD in U.S. whites was 14.4% (95% CI [14.0, 14.8]) [3]. Co-morbid conditions associated with NAFLD include obesity, type 2 diabetes (T2DM), hyperlipidemia, hypertension, and metabolic syndrome (MetS) [2].

Hemochromatosis in whites of western European descent is associated with homozygosity for *HFE* p.C282Y (rs1800562), a common missense allele of the homeostatic iron regulator (chromosome 6p22.2) in linkage disequilibrium with human leukocyte antigen (HLA)-A*03 [4, 5]. HFE, a non-classical class I major histocompatibility complex protein, is an upstream regulator of hepcidin and thus of iron homeostasis [6]. Laboratory phenotypes of many adults at diagnosis of hemochromatosis and p.C282Y/p.C282Y include elevated levels of transferrin saturation (TS) and serum ferritin (SF) [7]. Adults with p.C282Y/p.C282Y have increased risks to develop iron overload. Severe iron overload occurs predominantly in men [7, 8]. Non-*HFE* heritable and environmental variables modify iron loading in adults with hemochromatosis [5, 7, 9, 10]. Some adults with p.C282Y/p.C282Y also have hemochromatosis arthropathy, diabetes mellitus, hypogonadotropic hypogonadism, hepatic cirrhosis, or cardiomyopathy [7].

The aim of this study was to identify characteristics of non-alcoholic fatty liver disease (NAFLD) in adults with *HFE* p.C282Y/p.C282Y. We performed a retrospective study of clinical and laboratory characteristics of unrelated non-Hispanic white adults with hemochromatosis, iron overload, and p.C282Y/p.C282Y at non-screening diagnosis. The present probands, with or without NAFLD, did not report alcohol consumption > 14 g/d, have other liver disorders, report using steatogenic medication, or have diagnoses of heritable disorders that increase NAFLD risk. We identified significant NAFLD-associated characteristics using univariate and multivariable analyses. We discuss our findings in the context of previous observations of co-morbid conditions associated with NAFLD in adults who were and were not selected for *HFE* genotypes.

## Methods

### Subjects included

We compiled data on consecutive self-identified non-Hispanic whites aged ≥ 18 y referred to an Alabama tertiary hematology center (1989–2005) for evaluation and management of hemochromatosis who met the following criteria: (a) had *HFE* p.C282Y/p.C282Y, (b) had iron overload defined as serum ferritin (SF) > 300 µg/L (M) or > 200 µg/L (F) [11, 12], (c) underwent HLA-A typing, (d) had no known cause of secondary iron overload, (e) started and achieved iron depletion with therapeutic phlebotomy at this center, and (f) were the first in their respective families to be diagnosed to have hemochromatosis (probands).

Medical histories were taken from probands and records of referring physicians. Referring physicians diagnosed and treated probands with diabetes, hypertension, and hyperlipidemia. Physicians in the present hematology center evaluated probands for hemochromatosis arthropathy, hypogonadotropic hypogonadism, cirrhosis, and cardiomyopathy, as appropriate. All probands underwent medication review, physical examination, laboratory testing, imaging procedures, and evaluation of liver and other conditions, as indicated, before therapeutic phlebotomy was initiated.

### Subjects excluded

We excluded probands with any of the following: (a) diagnosis of hyperferritinemia, hemochromatosis, or *HFE* p.C282Y/p.C282Y in family or population screening, (b) diagnosis of any primary or secondary hematologic disorder, (c) report of alcohol consumption > 14 g/d drink-equivalent [13], (d) use of steatogenic medication(s) [14], (e) diagnosis of a heritable disorder that increases NAFLD risk [15, 16], (f) volunteer whole-blood donation > two units in the year before hemochromatosis diagnosis, (g) bariatric operations [17], (h) viral hepatitis B or C infection, (i) hepatic transient fibroelastography (FibroScan®, Echosens, Waltham, MA, USA) suggestive of severe hepatic fibrosis (≥ 9.4 kilopascals) or cirrhosis (≥ 11.0 kilopascals), (j) biopsy-proven cirrhosis, k) liver transplant, l) diagnosis of malignancy, m) anti-cancer therapy, n) chronic inflammatory conditions, or o) self-reported pregnancy.

### Laboratory

Blood specimens were collected during mornings without regard to fasting. TS and SF were measured using standard methods (Laboratory Corporation of America, Burlington, NC, USA). We defined these TS and SF phenotypes to be elevated: TS > 50% (men) and TS > 45% (women); and SF > 300 µg/L (men) and SF > 200 µg/L (women) [11, 12].

Serum alanine aminotransferase (ALT) and aspartate aminotransferase (AST) were measured using standard methods (Laboratory Corporation of America, Burlington, NC, USA). We defined upper limits of normal for ALT and AST as > 40 IU/L, respectively. We created a single dichotomous variable representing either elevated ALT or elevated AST (elevated ALT/AST).

*HFE* allele analyses were performed as previously described [18]. We demonstrated *HFE* p.C282Y homozygosity in 1996 or later in all probands regardless of dates of diagnoses. We defined HLA-A*03 as the marker of the hemochromatosis ancestral haplotype [18]. Positivity for A*03 was defined as A*03 homozygosity or heterozygosity.

### Liver biopsy

We recommended that probands undergo percutaneous liver biopsy (or not) in accordance with hemochromatosis diagnosis and management guidelines of the American Association for the Study of Liver Diseases [19].

### Diagnosis of non-alcoholic fatty liver disease

We diagnosed NAFLD according to the Practice Guideline of the American Association for the Study of Liver Diseases, American College of Gastroenterology, and the American Gastroenterological Association [20]. Diagnostic criterion was steatosis detected either by interpretation of liver biopsy specimens [1, 20], increased hepatic echogenicity [20, 21], or attenuation difference between liver and spleen on unenhanced CT scanning images [22], in the absence of self-report of alcohol consumption > 14 g/d drink-equivalent [13], cirrhosis or other non-NAFLD liver disorder, use of steatogenic medication [14], or diagnosis of a heritable disorder associated with increased NAFLD risk [15, 16].

### Definition of fibrosis-four variables and AST-to-platelet ratio indices

We computed the AST-to-platelet ratio index (APRI) [23] and fibrosis-four variables (FIB-4) index [24] in all probands. Threshold (or greater) values of each index are associated with increased risk for advanced hepatic fibrosis (stage F3, severe fibrosis with architectural distortion or stage F4, cirrhosis with architectural distortion) [25]. In a previous study of adults with hemochromatosis, APRI values > 3.25 had a positive predictive value for cirrhosis of 65% and a specificity of 97% [26]. FIB-4 index > 1.10 identified adults with *HFE* p.C282Y/p.C282Y with advanced fibrosis with 80% sensitivity, 80% specificity, and 81% accuracy [26].

### Definition of iron removed to achieve iron depletion

Iron depletion therapy, defined as the periodic removal of blood to eliminate storage iron, was performed in all probands as described elsewhere [27]. Iron depletion therapy was complete when SF was ≤ 20 µg/L [27]. Iron removed by phlebotomy to achieve iron depletion (QFe) was estimated to be 200 mg Fe per unit of blood (450–500 mL) [27].

### Definitions of other conditions

Definitions of hemochromatosis arthropathy, hypogonadotropic hypogonadism, cirrhosis, and cardiomyopathy are displayed in Additional file 1 [.docx; Other diagnostic criteria].

We defined obesity as ≥ 30.0 kg/m^2^ [28]. We classified diabetes according to criteria of the American Diabetes Association [29]. We grouped medical histories of essential hypertension or use of prescription anti-hypertensive drugs in a dichotomous hypertension variable. We grouped medical histories of hyperlipidemia or use of prescription lipid-lowering drugs in a dichotomous hyperlipidemia variable.

We defined metabolic syndrome (MetS) as the concurrence of the following three attributes: BMI ≥ 30 kg/m^2^; systolic blood pressure ≥ 130 mm Hg or diastolic blood pressure ≥ 85 mm Hg; and fasting serum glucose ≥ 100 mg/dL [28, 30, 31]. We grouped positivity for these three attributes in a dichotomous MetS variable.

### Statistics

The dataset for analyses consisted of complete observations at diagnosis of hemochromatosis, iron overload, and *HFE* p.C282Y/p.C282Y in 66 probands. Age, TS, and SF values are displayed to the nearest integer. Descriptive data are displayed as enumerations, percentages (with/without 95% CI), means (± 1 standard deviation (SD)), or medians (range).

Kolmogorov-Smirnov testing demonstrated that age and TS values did not differ significantly from those which are normally distributed. We displayed corresponding values as mean ± 1 SD and compared values using Student’s t test for unpaired data (two-tailed). We displayed other continuous variables as medians (range) and compared them using Mann-Whitney U test (two-tailed). We compared percentages using Fisher’s exact test (two-tailed). We did not use a Bonferroni “correction” for univariate comparisons because many data were not positively associated and we did not wish to produce “false negative” results [32]. We performed logistic regression on NAFLD by relaxing the “rule” of ten events per variable [33] and using independent variables identified in univariate comparisons for which “uncorrected” values of p were ≤ 0.1500 (SF, elevated ALT/AST, and T2DM).

We defined p < 0.05 to be significant. We used Excel® 2000 (Microsoft Corp., Redmond, WA, USA), GB-Stat® (v. 10.0, Dynamic Microsystems, Inc., Silver Spring, MD, USA), and GraphPad Prism 8® (2018; GraphPad Software, San Diego, CA, USA).

## Results

### General characteristics of probands

There were 31 men (47.0%) and 35 women (53.0%) of mean age 49 ± 14 y. NAFLD was diagnosed in 16 probands (24.2%; 95% CI [14.9, 36.6]). All probands underwent one or more imaging procedures that detect NAFLD [1, 20, 21]. Thirty-five probands (53.0%) underwent liver biopsy. Of 16 probands with NAFLD, 11 (68.8%) underwent liver biopsy. Of 50 probands without NAFLD, 24 (48.0%) underwent liver biopsy. These percentages did not differ significantly (p = 0.1653). Twenty-one probands (31.8%) underwent liver ultrasonography. Percentages of probands with and without NAFLD evaluated with liver ultrasonography did not differ significantly (25.0% vs. 34.0%, respectively; p = 0.5562). Eighteen probands (27.3%) underwent abdominal CT scanning. Percentages of probands with and without NAFLD evaluated with abdominal CT scanning did not differ significantly (18.8% vs. 30.0%, respectively; p = 0.5245). Eleven probands (16.7%) underwent liver evaluations with two of these modalities. Percentages of probands with and without NAFLD evaluated with two modalities did not differ significantly (25.0% vs. 14.0%, respectively; p = 0.4402). Two probands underwent fibroelastography as part of experimental or pre-marketing protocols (n = 2; 3.0%). Cirrhosis was not diagnosed in any proband based on clinical, liver biopsy, or imaging abnormalities.

Three of 16 probands (18.8%) with NAFLD had none of the NAFLD co-morbid conditions we studied. Median SF and prevalences of elevated ALT/AST and T2DM were greater in probands with than without NAFLD (Table 1).

Hemochromatosis arthropathy was diagnosed in 24 probands (36.4%). The prevalence of hemochromatosis arthropathy did not differ significantly in probands with and without NAFLD (31.3% vs. 38.3%, respectively; p ~ 1.0000). No proband was diagnosed to have hypogonadotropic hypogonadism, cirrhosis, or cardiomyopathy.

**Table 1:** Characteristics of hemochromatosis probands with *HFE* p.C282Y/p.C282Y^a^

Characteristic	NAFLD (n = 16)	No NAFLD (n = 50)	Value of p
Age at diagnosis, y (± 1 SD)	51 ± 13	49 ± 15	0.6614
Men, % (n)	56.3 (9)	44.0 (22)	0.5659
Mean transferrin saturation, % (± 1 SD)	77 ± 14	83 ± 17	0.1626
Median serum ferritin, µg/L (range)	1118 (259, 2663)	567 (247, 2385)	0.0192
HLA-A*03 positivity, % (n)	75.0 (12)	82.0 (41)	0.7187
Elevated ALT/AST, % (n)	43.8 (7)	10.0 (5)	0.0056
Obesity (BMI ≥ 30.0 kg/m^2^), % (n)	18.9 (3)	16.0 (8)	~ 1.0000
Diabetes, type 2, % (n)	31.3 (5)	10.0 (5)	0.0427
Hypertension, % (n)	25.0 (4)	18.0 (9)	0.7187
Hyperlipidemia, % (n)	6.3 (1)	12.0 (6)	1.0000
Metabolic syndrome, % (n)	0.0 (0)	4.0 (2)	~ 1.0000

^a^ All observations were recorded at diagnosis. Blood specimens were collected during mornings without regard to fasting. ALT, alanine aminotransferase; AST, aspartate aminotransferase; BMI, body mass index; HLA, human leukocyte antigen; NAFLD, non-alcoholic fatty liver disease; SD, standard deviation. No proband had hypogonadotropic hypogonadism, cirrhosis, or cardiomyopathy.

The prevalence of elevated ALT/AST was significantly greater in probands with than without NAFLD (Table 1). The prevalence of elevated ALT was greater in probands with than without NAFLD (37.5% vs. 8.0%, respectively; p = 0.0009). The prevalence of elevated AST was greater in probands with than without NAFLD (43.8% vs. 4.0%, respectively; p = 0.0004). Nine of 16 probands with NAFLD (56.3%) did not have elevated ALT or AST.

No proband had APRI > 0.44, although median APRI was greater in probands with than without NAFLD (0.15 (range 0.07–0.37) vs. 0.10 (range 0.04–0.23); p = 0.0025). Median FIB-4 in probands with and without NAFLD did not differ significantly (1.10 (range 0.53–3.29) vs. 0.99 (range 0.28–4.99); p = 0.2915). One woman with and another woman without NAFLD had FIB-4 > 3.25, although their respective liver biopsy specimens did not reveal cirrhosis.

### Co-morbid conditions associated with non-alcoholic fatty liver disease

The prevalence of T2DM was greater in probands with than without NAFLD (Table 1). Respective prevalences of obesity, hypertension, hyperlipidemia, and MetS in probands with and without NAFLD did not differ significantly (Table 1).

### Logistic regression on non-alcoholic fatty liver disease

Logistic regression on NAFLD, the dependent variable, using the qualifying independent variables SF, elevated ALT/AST, and T2DM, revealed two positive associations: SF (p = 0.0318; beta coefficient 0.0011; standard error 0.0005) and T2DM (p = 0.0342; beta coefficient 1.5973; standard error 0.7543). This regression (ANOVA p = 0.0362) accounted for 11.6% of the variance of NAFLD. Odds ratios were SF (1.000-1.002) and T2DM (1.094–22.311).

### Iron removed by phlebotomy to achieve depletion

Median QFe in probands with and without NAFLD did not differ significantly (3.6 g (1.4–7.2) vs. 2.8 g (0.7–11.0), respectively; p = 0.6862).

## Discussion

The present study evaluated clinical and laboratory associations of NAFLD in a cohort of non-screening adults with hemochromatosis, iron overload, and *HFE* p.C282Y/p.C282Y who did not report alcohol consumption > 14 g/d, have cirrhosis or other liver disorders, report using steatogenic medication, or have diagnoses of heritable disorders that increase NAFLD risk. The prevalence of NAFLD in the present cohort was 24.2% (95% CI [14.9, 36.6]). In a meta-analysis of 43 studies with 5,758 NAFLD cases and 14,741 controls from diverse geographic regions, “a significantly increased risk of NAFLD was observed for the C282Y polymorphism in the Caucasian population under all genetic models [34].“ In the same meta-analysis, NAFLD risk in adults with p.C282Y/p.C282Y and adults with wt/wt (absence of p.C282Y and p.H63D (rs1799945)) did not differ significantly [34].

Median SF was almost two-fold greater in the present probands with than without NAFLD. In patients with hemochromatosis and *HFE* p.C282Y homozygosity, there was is a significant positive correlation of phlebotomy-mobilized iron with hepatic iron concentration [35], although QFe in the present study did not differ significantly in probands with and without NAFLD. In another study, median SF level but not hepatic iron concentration was significantly higher in p.C282Y homozygotes with than without hepatic steatosis [36]. Together, these observations indicate that NAFLD contributes significantly to hyperferritinemia but not iron overload in p.C282Y homozygotes.

Prevalences of elevated ALT and elevated AST were greater in the present probands with than without NAFLD, although 56.3% of probands with NAFLD had neither elevated ALT nor elevated AST, and elevated ALT/AST was not significantly associated with NAFLD in a logistic regression. In a meta-analysis of 11 studies of patients unselected for *HFE* genotypes, 25% of patients with NAFLD and 19% of patients with non-alcoholic steatohepatitis had ALT values within the reference range [37]. These observations suggest that evaluation for NAFLD should be considered at diagnosis in all subjects with hemochromatosis and *HFE* p.C282Y/p.C282Y, regardless of ALT and AST levels, although current guidelines for hemochromatosis diagnosis and management do not recommend evaluation for NAFLD in all patients with or suspected to have hemochromatosis [19].

The prevalence of obesity (BMI ≥ 30 kg/m^2^) in the present cohort (15.2% (95% CI [7.9–26.6]) did not differ significantly in probands with and without NAFLD. We found no reports of the prevalence of obesity in screening programs that evaluated participants with *HFE* p.C282Y/p.C282Y. It is estimated that the worldwide prevalence of obesity in subjects with NAFLD is ~ 51% [2].

In a Utah study [38], white men with *HFE* p.C282Y/p.C282Y aged ≥ 40 y had significantly lower mean BMI (26.7 ± 0.5 kg/m^2^) than control siblings without p.C282Y/p.C282Y (30.5 ± 1.6 kg/m^2^) and male 1999–2002 National Health and Nutrition Examination Survey participants (28.7 ± 0.3 kg/m^2^) [38]. Corresponding comparisons in women were not significant [38]. In a U.S. atherosclerosis risk screening program, median BMI did not differ significantly in 44 white participants with p.C282Y/p.C282Y (26.5 kg/m^2^ (standard error (SE) 0.1)) and 6768 white participants with wt/wt (26.9 kg/m^2^ (SE 0.1)) [39]. Thus, it is uncertain whether BMI in adults with p.C282Y/p.C282Y differs from that in adults in the general U.S. population.

Prevalence of T2DM was greater in the present probands with than without NAFLD. T2DM was also significantly associated with NAFLD, after adjustment for other variables. These observations suggest that presence of T2D at diagnosis in hemochromatosis and *HFE* p.C282Y/p.C282Y should prompt evaluations for NAFLD. In a study of 159 non-screening Alabama adult hemochromatosis probands with *HFE* p.C282Y/p.C282Y, predictors of T2DM at hemochromatosis diagnosis were diabetes reports in first-degree family members (odds ratio 8.5 (95% CI [2.9, 24.8])) and BMI ≥ 30.0 kg/m^2^ (odds ratio 1.1 (95% CI [1.0, 1.2])). In another study, NAFLD was also a significant risk factor for concurrent or future T2DM diagnoses in adults unselected for *HFE* genotypes in the general population [40, 41]. Together, these observations indicate that NAFLD, diabetes reports in first-degree family members, and obesity are associated with T2DM in adults with p.C282Y/p.C282Y.

The prevalence of hypertension in the present cohort was 19.7% (95% CI [11.3, 31.7]). The prevalence of hypertension in probands with and without NAFLD did not differ significantly, although NAFLD is a possible risk factor for hypertension [42, 43]. In studies of Scandinavian population cohorts, *HFE* p.C282Y/p.C282Y and extremely elevated TS, either separately or combined, were associated with increased risk of anti-hypertension medication use [44].

The prevalence of hyperlipidemia in the present cohort was 10.6% (95% CI [4.7, 21.2]). The prevalence of hyperlipidemia in the present probands with and without NAFLD did not differ significantly. In an atherosclerosis risk screening study, mean low-density lipoprotein (LDL) cholesterol was lower in participants with *HFE* p.C282Y/p.C282Y than wt/wt [39]. In a primary care-based hemochromatosis and iron overload screening study, participants with p.C282Y/p.C282Y had lower total and LDL cholesterol levels than participants with wt/wt [45]. It has been postulated that lower LDL cholesterol in adults with p.C282Y/p.C282Y is an effect of excess iron on cholesterol metabolism and lipoprotein formation in the liver [39]. Triglyceride levels in subjects with p.C282Y/p.C282Y and wt/wt in two population screening programs did not differ significantly [39, 45].

MetS was uncommon in the present cohort (3.0% (95% CI [0.5, 11.5])) and the prevalence of MetS in probands with and without NAFLD did not differ significantly. In a post-screening evaluation of 248 participants with *HFE* p.C282Y/p.C282Y, the prevalence of MetS was also relatively low 7.3% (95% CI [4.5, 11.4]) [46]. We attribute the relatively low prevalence of MetS in the present cohort to relatively low prevalences of obesity, hypertension, and hyperlipidemia.

Probands with cirrhosis were excluded from the present study, although < 8% of adults with *HFE* p.C282Y/p.C282Y diagnosed in either screening or non-screening cohorts published in the 21st century have cirrhosis [47]. In a multi-institutional study, cirrhosis in patients with p.C282Y/p.C282Y was significantly associated with age, diabetes, daily alcohol intake, and iron removed by phlebotomy, taking into account the effect of other variables, although the prevalence of fatty liver/steatosis did not differ significantly in 86 adults with and 282 adults without cirrhosis (27.4% and 33.0%, respectively; p = 0.4400) [8]. In a large study of healthcare records in UK, Netherlands, Italy and Spain, the hazard ratio for cirrhosis in patients with NAFLD was 4.73 (95% CI [2.43, 9.19]), although the strongest independent predictor of cirrhosis was a baseline diagnosis of diabetes [48]. In a study that did not include subjects with p.C282Y/p.C282Y, SF, age, BMI, and diabetes were independent predictors of histologic severity and advanced fibrosis in patients with NAFLD [49]. Together, these observations suggest that age and diabetes are greater risk factors for cirrhosis in adults with p.C282Y/p.C282Y than NAFLD.

In summary, the percentage of the present hemochromatosis probands with NAFLD (24.2%; 95% CI [14.9, 36.6]) was higher than that of whites in general U.S. populations (14.4%; 95% CI [14.0, 14.8]) [3]. In a meta-analysis of global populations, co-morbid conditions associated with NAFLD included obesity, T2DM, hyperlipidemia, hypertension, and MetS [2], whereas T2D alone was significantly associated with NAFLD in the present hemochromatosis probands. Referring physicians diagnosed and treated the present probands with diabetes, hypertension, and hyperlipidemia and thus their evaluations and diagnostic criteria for these disorders may differ from those of large population studies. LDL cholesterol [39, 45] and total cholesterol [45] levels are lower in adults with *HFE* p.C282Y/p.C282Y than wt/wt. These factors could account in part for the absence of NAFLD risk factors we studied in 18.8% of the present probands. It is also plausible that probands without NAFLD risk factors we studied have other undiagnosed or undocumented conditions that increase NAFLD risk, including obstructive sleep apnea [50], hypothyroidism [51, 52], hypopituitarism [53], or heritable disorder(s) [15, 16].

A strength of the present study is analyses based on observations of non-screening adults with hemochromatosis, iron overload, and *HFE* p.C282Y/p.C282Y, with or without NAFLD, who did not report alcohol consumption > 14 g/d, have cirrhosis or other liver disorders, report using steatogenic medication, or have diagnoses of heritable disorders that increase NAFLD risk. The present study does not permit a comparison of the sensitivity and specificity of liver biopsy, ultrasonography, and CT scanning in diagnosing NAFLD. It is also plausible that the lack of significant difference of the prevalence of NAFLD co-morbid factors other than T2DM we studied between probands with and without NAFLD may in part reflect type 2 statistical error(s). Evaluating subgroups of probands with NAFLD based on ALT/AST values or liver morphology [2] or alcoholic/non-alcoholic fatty liver scores/indices, detecting alleles associated with increased NAFLD risk [15, 16], determining the effects of therapeutic phlebotomy on ALT and AST levels, treating NAFLD, and evaluating of post-diagnosis observations other than QFe were beyond the scope of this work.

## Conclusions

We conclude that NAFLD in hemochromatosis probands with iron overload and *HFE* p.C282Y/p.C282Y is associated with higher median SF and greater T2DM prevalence, after adjustment for other factors. NAFLD does not influence QFe significantly.

## Electronic supplementary material

Below is the link to the electronic supplementary material.

Supplementary Material 1. Definitions of other conditions

## Data Availability

The dataset generated and/or analysed during the current study is available in the Open Science Framework repository (https://osf.io/j5gsp/). Data were compiled and displayed in a manner that maintains proband anonymity. Definitions of hemochromatosis arthropathy, hypogonadotropic hypogonadism, cirrhosis, and cardiomyopathy are displayed in Additional file 1 [.docx; Other diagnostic criteria].

## List of abbreviations

ALD: alcoholic liver disease
ALT: alanine aminotransferase
ANI: alcoholic liver disease/non-alcoholic fatty liver disease index
APRI: AST-to-platelet ratio index
AST: aspartate aminotransferase
BMI: body mass index
CI: confidence interval
FIB-4: fibrosis-four variables
*HFE*: homeostatic iron regulator gene
HLA: human leukocyte antigen
LDL: low-density lipoprotein
MetS: metabolic syndrome
NAFLD: non-alcoholic fatty liver disease
QFe: quantity of iron removed by phlebotomy to achieve iron depletion
SD: standard deviation
SE: standard error
SF: serum ferritin
T2DM: type 2 diabetes mellitus
TS: transferrin saturation
