# Supplementary material for: Non-alcoholic fatty liver disease in hemochromatosis probands with iron overload and HFE p.C282Y/p.C282Y
Source: BMC Gastroenterol. 2023 Apr 28;23:137. doi: 10.1186/s12876-023-02763-x (PMC10148383; doi:10.1186/s12876-023-02763-x)
Supplement: Supplementary file 1 — Supplementary Material 1. Definitions of other conditions [file 12876_2023_2763_MOESM1_ESM.docx]

**Definitions of other conditions**

*Hemochromatosis arthropathy*

We defined hemochromatosis hand arthropathy as the combination of tenderness, pain, swelling, or effusion of the second and third metacarpophalangeal joints [1,2] and radiological features consistent with those observed in hemochromatosis [3] in the absence of other cause(s). We defined hemochromatosis non-hand arthropathy as physical examination and radiological abnormalities in shoulders, elbows, hips, knees, and ankles typical of hemochromatosis [3] in the absence of other cause(s). We classified probands who had either hemochromatosis hand arthropathy, hemochromatosis non-hand arthropathy, or both as having hemochromatosis arthropathy. We did not classify autoimmune or neuropathic arthropathy as hemochromatosis arthropathy. We did not evaluate observations on spine or other arthropathy. The present probands with hemochromatosis arthropathy were referred to consulting rheumatologists.

*Hypogonadotropic hypogonadism*

Serum levels of total testosterone, luteinizing hormone, and follicle-stimulating hormone were measured at diagnosis of hemochromatosis in men who reported loss of libido or erectile dysfunction due to undefined cause(s) and did not take supplemental androgen(s). Serum levels of estradiol, luteinizing hormone, and follicle-stimulating hormone were measured at diagnosis of hemochromatosis in women who reported premature cessation of menses not due to menopause or other defined cause(s) and did not take hormone replacement therapy. Hypogonadotropic hypogonadism was defined as the combination of subnormal serum total testosterone levels <241 ng/dL (<8.4 nmol/L) (men) or serum estradiol levels <30 pg/mL (110 pmol/L) (women) and serum luteinizing hormone and follicle-stimulating hormone levels that did not exceed the upper reference limits (9.3 IU/L and 18.1 IU/L, respectively), in the absence of other cause(s) [4].

*Cirrhosis*

We recommended that probands undergo percutaneous liver biopsy (or not) in accordance with hemochromatosis diagnosis and management guidelines of the American Association for the Study of Liver Diseases [5]. Iron staining in liver specimens was graded 0-4+ as previously described [6]. Hepatic iron was measured using atomic absorption spectrometry. Pathologists interpreted liver specimens and defined cirrhosis as the histological occurrence of regenerating nodules of hepatocytes surrounded by bands of fibrous connective tissue [7]. In some probands, the presence or absence of cirrhosis was determined using imaging techniques (CT scanning, abdominal ultrasonography, or fibroelastography) [8].

*Cardiomyopathy*

Hemochromatosis cardiomyopathy was defined as dilated cardiomyopathy with low left ventricular ejection fraction and decreased fractional shortening, with or without complete atrioventricular block or atrial and ventricular tachyarrhythmias, due to cardiac siderosis demonstrated by endomyocardial biopsy or MRI scanning [3,9].

References

1. Allen KJ, Gurrin LC, Constantine CC, Osborne NJ, Delatycki MB, Nicoll AJ, et al. Iron-overload-related disease in *HFE* hereditary hemochromatosis. N Engl J Med. 2008;358:221-30.

2. McLaren GD, McLaren CE, Adams PC, Barton JC, Reboussin DM, Gordeuk VR, et al. Clinical manifestations of hemochromatosis in *HFE* C282Y homozygotes identified by screening. Can J Gastroenterol. 2008;22:923-30.

3. Edwards CQ, Barton J.C. Hemochromatosis. In: Greer JP, Arber DA, Glader B, List AF, Means Jr. RT, Paraskevas F, et al., editors. Wintrobe's Clinical Hematology. Philadelphia: Wolters Kluwer/Lippincott Williams & Wilkins; 2014. p. 662-681.

4. McDermott JH, Walsh CH. Hypogonadism in hereditary hemochromatosis. J Clin Endocrinol Metab. 2005;90:2451-5.

5. Bacon BR, Adams PC, Kowdley KV, Powell LW, Tavill AS. Diagnosis and management of hemochromatosis: 2011 Practice Guideline by the American Association for the Study of Liver Diseases. Hepatology. 2011;54:328-43.

6. Scheuer PJ, Williams R, Muir AR. Hepatic pathology in relatives of patients with haemochromatosis. J Pathol Bacteriol. 1962;84:53-64.

7. McCormick PA: Hepatic cirrhosis. In: Dooley JS, Lok ASF, Burroughs AK, Heathcote EJ, editors. Sherlock's Diseases of the Liver and Biliary System. Chichester: Wiley-Blackwell; 2012. p. 103-120.

8. Procopet B, Berzigotti A. Diagnosis of cirrhosis and portal hypertension: imaging, non-invasive markers of fibrosis and liver biopsy. Gastroenterol Rep (Oxf). 2017;5:79-89.

9. Aronow WS: Management of cardiac hemochromatosis. Arch Med Sci. 2018;14:560-8.
